# Alleviating effect of *Lactobacillus fermentum* E15 on hyperlipidemia and hepatic lipid metabolism in zebrafish fed by a high-fat diet through the production of short-chain fatty acids

**DOI:** 10.3389/fnut.2025.1522982

**Published:** 2025-03-03

**Authors:** Yishu Chen, Kangdi Zheng, Yang Leng, Zhao Zhang, Xiaoling Li, Xiaoyan Li, Huajun Ou, Muhao Wen, Feng Qiu, Huajun Yu

**Affiliations:** ^1^Laboratory Animal Center, Guangdong Medical University, Zhanjiang, China; ^2^Guangdong Longseek Testing Co., Ltd., Guangzhou, China; ^3^Department of Laboratory Medicine, the Seventh Affiliated Hospital of Southern Medical University, Foshan, China

**Keywords:** *Lactobacillus fermentum* E15, short-chain fatty acids, hyperlipidemia, high-cholesterol diet, zebrafish

## Abstract

**Introduction:**

Hyperlipidemia is regarded as one of the crucial factors leading to atherosclerosis and other cardiovascular diseases. Gut microbiota plays an important role in regulating host lipid metabolism. Nevertheless, the exact mechanisms behind this remain unclear.

**Methods:**

In the present study, a hyperlipidemic zebrafish model was established using a high-cholesterol diet (HCD) to evaluate the anti-hyperlipidemic effects of *Lactobacillus fermentum* E15 (*L. fermentum* E15).

**Results:**

Results showed that *L. fermentum* E15 effectively reduced lipid accumulation in the blood vessels and liver of HCD-fed zebrafish larvae. Meanwhile, *L. fermentum* E15 improved abnormal lipid levels, and normalized liver enzyme activity. Real-time quantitative polymerase chain reaction (RT-qPCR) analysis revealed that *L. fermentum* E15 downregulated the expression of sterol regulatory element-binding factor (SREBP-1), peroxisome proliferator-activated receptor-gamma (PPAR-*γ*), and fatty acid synthase (Fasn), while upregulated peroxisome proliferator-activated receptor-alpha (PPAR-*α*). Additionally, metabolomic analysis revealed that *L. fermentum* E15 produced a series of short-chain fatty acids (SCFAs), including acetic acid, propionic acid, butyric acid, and isovaleric acid. Notably, isovaleric acid contributed to the reduction of lipid droplet accumulation in the liver and blood vessels of HCD-fed zebrafish larvae. In contrast, blocking G-protein coupled receptor 43 (GPR43) with pertussis toxin (PTX) abolished the effects of *L. fermentum* E15 and isovaleric acid on reducing lipid accumulation in HCD-fed zebrafish larvae. RT-qPCR results further suggested that both *L. fermentum* E15 and isovaleric acid promoted the expression of GPR43 and leptin A, which was inhibited by PTX.

**Conclusion:**

These findings suggested that *L. fermentum* E15 alleviates HCD-induced hyperlipidemia by activating GPR43 through SCFAs.

## 1 Introduction

Hyperlipidemia is a common lipid metabolism disorder, which is primarily characterized by elevated levels of total cholesterol (TC), triglycerides (TG), low-density lipoprotein cholesterol (LDL-C) and low level of high-density lipoprotein cholesterol (HDL-C) (1). Numerous evidences have shown that hyperlipidemia can cause atherosclerosis, increasing the risk of stroke, hypertension and coronary heart disease, thereby threating human health (2, 3). The liver is a key organ in lipid metabolism involved in a series of physiological activities, including digestion, absorption, transport, degradation and synthesis of lipids (4, 5). Previous study has reported that lipid accumulation in liver can induce hepatotoxicity and inflammatory responses, thereby exacerbating lipid metabolism disorder (6). However, the etiology of hyperlipidemia is complex, and the underlying mechanisms are still required to be fully clarified. Gut microbiota has been associated with hyperlipidemia due to its regulatory role in the storage, degradation and distribution of lipids (7–10). Thus, targeting the gut microbiota is a potential effective strategy for hyperlipidemia treatment.

Probiotics have been applied to alleviate and treat metabolic diseases induced by a high-fat diet (11). Supplementation of a mix of *Bifidobacterium animalis* subsp. lactis LA804 and *Lactobacillus gasseri* LA806 has been reported to reduce body weight and fat tissue accumulation in mice, simultaneously reducing the plasma triglyceride level, hepatic lipid accumulation, and preventing inflammation (12). Besides, under the hypercholesterolemia condition, the colonization of *Lactobacillus plantarum* in rats has been shown to reduce TC, TG, LDL-C, alanine aminotransferase (ALT), aspartate aminotransferase (AST), very-low-density lipoprotein, and atherosclerotic index in the serum (13). In another study by Park et al. regarding the effects of *Lactobacillus plantarum* in a human clinical trial, *Lactobacillus plantarum* Q180 was found to decrease the maximum postprandial concentration levels of TG, LDL-C, apolipoprotein B-100, and apolipoprotein B-48 (14). Nevertheless, the mechanisms of action of probiotics require further investigation. Microbiota-driven metabolites including short-chain fatty acids (SCFAs), lipopolysaccharides, and bile acids have been reported to act as key signal transduction molecules that couple gut microbiota with the host (7, 15–17). Many studies have demonstrated that microbial metabolite SCFAs can coordinate signals involved in the lipid metabolism and inflammatory physiological regulation of host through mediating G protein-coupled receptors (GPRs) in gut or liver (7, 15, 18, 19). Zebrafish are small freshwater fish which have been proposed to be an emerging and promising animal model for drug screening due to the unique advantages of small size, transparent embryos and short experimental cycles as compared to the mammalian models (20). The lipid metabolism in zebrafish is similar to that of humans, such as intestinal lipid absorption and lipoprotein-mediated cholesterol transport (21), making zebrafish an excellent model for studying lipid metabolism-related diseases (22–24).

In this study, our objective is to evaluate the potential hypolipidemic effect of *L. fermentum* E15 and the mechanism underlying such an effect. For this purpose, zebrafish were fed a HCD supplemented with *L. fermentum* E15. Zebrafish larvae were stained using Oil Red O and hematoxylin and eosin (H&E) to evaluate lipid deposition after 26 days of incubation. In addition, lipid (TG, TC, LDL-C, and HDL-C) concentrations, liver enzyme (AST and ALT) activities, and the level of oxidative stress were analyzed using relevant assay kits. Furthermore, the content of SCFAs produced by *L. fermentum* E15 was assessed using liquid chromatography tandem mass spectrometry (LC–MS/MS) technology. To verify the hypothesis that the anti-hyperlipidemic effects of *L. fermentum* E15 occur through SCFAs, we blocked GPR43 in zebrafish larvae by using PTX, an antagonist of GPRs, to investigate the alleviating effects of *L. fermentum* E15 and isovaleric acid on lipid metabolism disorders. In addition, the expression levels of key genes involved in lipid metabolism were evaluated using RT-qPCR. The obtained results provide theoretical evidence for the development of *L. fermentum* E15 dietary supplements.

## 2 Materials and methods

### 2.1 Materials and reagents

In this experiment, wild-type AB strain zebrafish were purchased from the National Zebrafish Resource Center (Wuhan, China). *L. fermentum* E15 was isolated from the feces of healthy infants and preserved at the China General Microbiological Culture Collection Center (CGMCC) with the accession number 22009.

De-Man Rogosa and Sharpe (MRS) medium was purchased from Qingdao Hi-Tech Industrial Park Hope Bio-Technology Co., Ltd. (Qingdao, China). The RNA rapid extraction kit and FastQuant RT Kit (with gDNase) were obtained from Tiangen Biotech Co., Ltd. (Beijing, China). Sodium isovalerate was purchased from Shanghai Yuanye Bio-Technology Co., Ltd. (Shanghai, China). Commercial assay kits for total protein (BCA), triglycerides (TG), total cholesterol (TC), low-density lipoprotein cholesterol (LDL-C), high-density lipoprotein cholesterol (HDL-C), aspartate aminotransferase (AST), alanine aminotransferase (ALT), superoxide dismutase (SOD) and malondialdehyde (MDA) were purchased from Nanjing Jiancheng Bioengineering Institute (Nanjing, China). Cholesterol, pertussis toxin (PTX), 2′,7′-dichlorodihydrofluorescein diacetate (DCFH-DA), and Oil Red O were purchased from Sigma-Aldrich. All reagents of high-performance liquid chromatography (HPLC) grade and short-chain fatty acid metabolite standards were purchased from Sigma-Aldrich.

### 2.2 Morphological identification of *Lactobacillus fermentum* E15

To observe the macroscopic morphology of *L. fermentum* E15, the strain was streaked onto MRS solid medium and incubated at 37°C for 48 h in an anaerobic workstation (E500G, GeneScience, United States). The strain was then identified using Gram staining.

### 2.3 Cultivation and preparation of *Lactobacillus fermentum* E15

*Lactobacillus fermentum* E15 was inoculated in MRS medium (5 mL liquid medium, autoclaved at 121°C for 15 min) and cultured at 37°C with gentle shaking at 100 rpm for 24 h. Following this, the inoculum was prepared by inoculating 1 mL of the culture to a 50 mL centrifuge tube containing 40 mL of MRS broth medium. The centrifuge tube was then incubated at 37°C and 100 rpm for 24 h. After incubation, bacterial pellet was collected by centrifuging at 10000 rpm for 5 min, followed by two washes with phosphate-buffered saline (PBS) and resuspension in E3 water (5 mM NaCl, 0.17 mM KCl, 0.33 mM CaCl₂, and 0.33 mM MgSO₄). The bacterial pellet was adjusted to final concentrations of 1 × 10^4^ CFU/mL, 1 × 10^5^ CFU/mL, and 1 × 10^6^ CFU/mL, respectively.

### 2.4 Preparation of high cholesterol diet

The normal diet (AP100) for zebrafish larvae supplemented with 12% crude fat and 50% crude protein was purchased from Zeigler (Pennsylvania, United States). After the mixture of cholesterol ether solution with the normal diet, the ether was evaporated to obtain the HCD diet and the final concentration of cholesterol in the HCD diet was determined to be 4% (w/w).

### 2.5 Maintenance and treatment of zebrafish larvae

**Figure 1:**
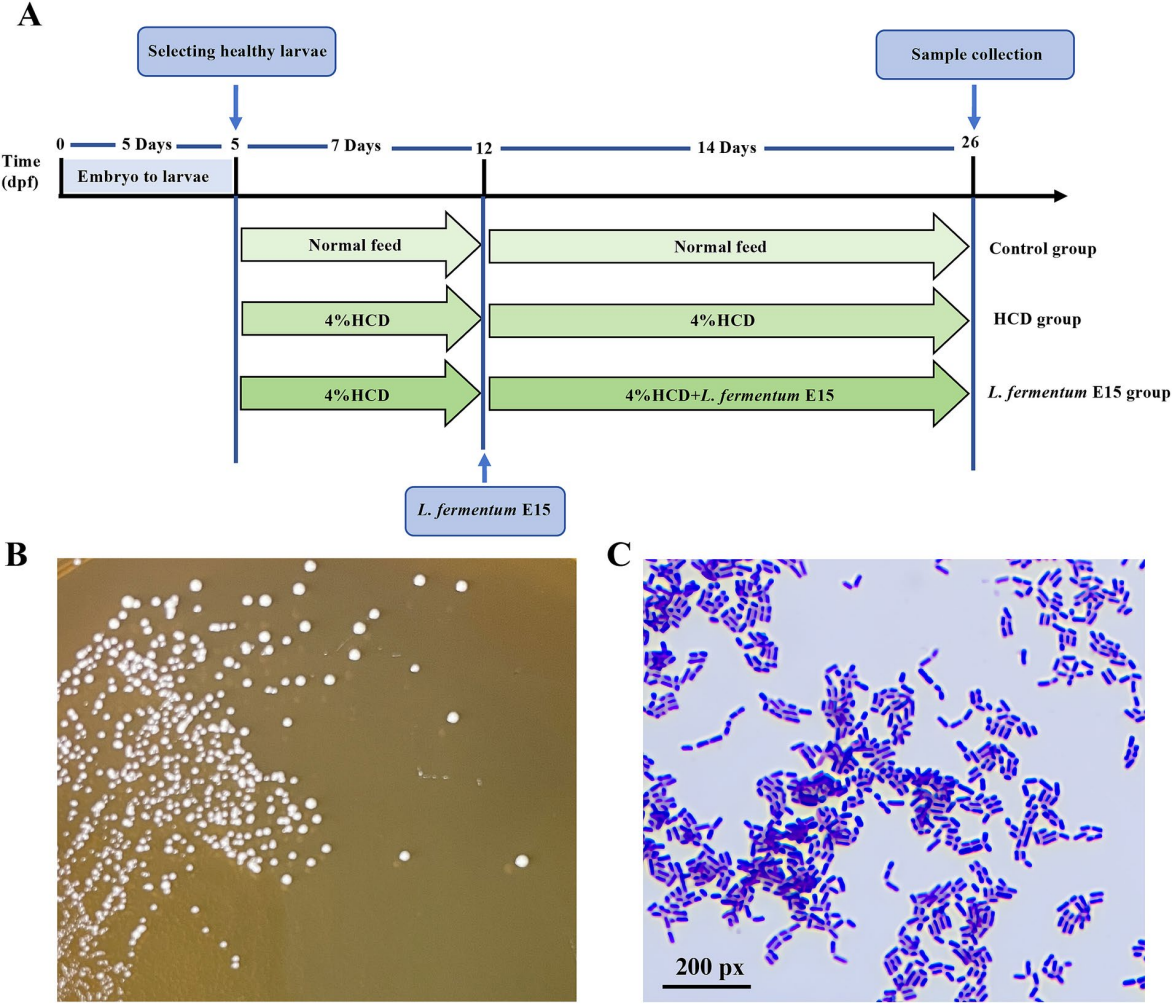
Experiment outline and Characterization of *L*. *fermentum* E15. **(A)** Experimental outline of *L. fermentum* E15 treated zebrafish larval fed by HCD. **(B)** *L*. *fermentum* E15 colonies on MRS agar cultured anaerobically after 48 h at 37°C. **(C)** Gram staining properties of *L*. *fermentum* E15.

Wild-type AB strain adult zebrafish were maintained in a zebrafish breeding system (28.5°C, pH 7.5, conductivity 500–550 μS/cm, 14:10 h light–dark cycle). Afterwards, zebrafish embryos were obtained from those adult zebrafish through natural mating, and then incubated in E3 water (5 mM NaCl, 0.17 mM KCl, 0.33 mM CaCl_2_, and 0.33 mM MgSO_4_) and cultured in an incubator at a constant temperature of 28°C. Five days post fertilization (dpf) wild-type AB strain zebrafish larvae were randomly divided into 5 groups with 100 zebrafish larvae in each group, followed by incubation in a 1 L water tank. There were 5 treatments, namely, normal diet (control), 4% HCD (HCD group), 4% HCD + 1 × 10^4^ CFU/mL *L. fermentum* E15 (1 × 10^4^ CFU/mL *L. fermentum* E15 group), 4% HCD + 1 × 10^5^ CFU/mL *L. fermentum* E15 (1 × 10^5^ CFU/mL *L. fermentum* E15 group), or 4% HCD + 1 × 10^6^ CFU/mL *L. fermentum* E15 (1 × 10^6^ CFU/mL *L. fermentum* E15 group). After 5 dpf, the control group was fed with a normal diet. The HCD group and the *L. fermentum* E15 group were fed with a HCD for a period of 7 days (5–12 dpf). In the following 14-day period (12–26 dpf), the control group continued to be fed with a normal diet, while the HCD group continued to be fed with a HCD. Meanwhile, the *L. fermentum* E15 group was fed with a HCD supplemented with *L. fermentum* E15 for 14 days (12–26 dpf). All the groups were fed with the same amount of feed 30 mg/d (twice a day), as described in Figure 1A, and the residual food was removed after 1 h of feeding. All the zebrafish experiments were approved by Animal Welfare and Ethics Committee of Guangdong Human Microecology Engineering Technology Research Center’s Laboratory (approval number: IACUC MC 0329-01-2024).

### 2.6 Oil red O staining

After the intervention, 20 zebrafish larvae were randomly collected from each group, washed twice with E3 water, and fixed in 4% paraformaldehyde solution for 24 h. These zebrafish larvae were subjected to 25, 50, 75, and 100% 1,2-propanediol gradient dehydration with each gradient for 25 min. Afterwards, these zebrafish were stained with Oil Red O for 48 h and then destained with 1,2-propanediol for 30 min, followed by two washes with PBS. A stereomicroscope (SZ680) was used to capture the images and analyze the lipid accumulation in the zebrafish. The integrated optical density (IOD) values were quantified using ImageJ software to determine the relative quantitative index of lipid accumulation.

### 2.7 Analysis of body mass index

After 14 days of treatment with *L. fermentum* E15, 20 zebrafish larvae were randomly selected from each group and anesthetized using tricaine (0.1 g/L). The body length of zebrafish larvae was measured using a stereomicroscope (SZ680), while the weight of zebrafish larvae was measured using an analytical balance. Subsequently, the BMI value (mg/mm^2^) was calculated as the ratio of the measured weight to body length.

### 2.8 Biochemical analysis

For each group, 10 zebrafish larvae were randomly selected as one sample, and 3 samples were prepared for subsequent assessments. Briefly, zebrafish larvae were anesthetized in a tricaine solution (0.1 g/L) and subsequently euthanized by immersion in ice-cold E3 water bath as previously described (25). Afterwards, the larvae were washed with pre-chilled PBS and then homogenized in 500 μL of physiological saline. The supernatant was collected by centrifugation at 4°C and 13,000 rpm g for 10 min. The levels of TG, TC, LDL-C, HDL-C, AST activity, ALT activity, SOD activity and MDA were measured using commercial assay kits (Nanjing Jiancheng Bioengineering Institute, China) following the manufacturer’s instructions.

### 2.9 Hematoxylin and eosin staining

The zebrafish larvae were fixed with 4% paraformaldehyde solution for 24 h before being processed according to standard procedures for H&E staining. Afterwards, the zebrafish larvae were embedded in paraffin then sectioned and stained with H&E. Microscopy was performed to observe the pathological changes in zebrafish liver tissue using a microscope (Nikon Eclipse E100).

### 2.10 Measurement of reactive oxygen species

The ROS levels in zebrafish larvae were assessed using the fluorescent probe dye DCFH-DA (Sigma-Aldrich). Fourteen days after the treatment with *L. fermentum* E15, 10 zebrafish larvae were randomly collected from each group and placed in a 6-well cell culture plate with 5 mL DCFH-DA (5 μM) solution into each well. All the samples were incubated in the dark at 28.5°C for 1 h followed by three washes with E3 water and observation under a fluorescence microscope (Leica DMi8). The statistical analysis of fluorescence intensity of individual zebrafish larvae was performed using the ImageJ software.

### 2.11 Real-time quantitative PCR analysis

After the intervention, 20 zebrafish larvae were randomly selected from each group and then subjected to euthanasia for total RNA extraction using Trizol reagent (Invitrogen, United States) after the intervention. Reverse transcription was conducted using HiScript II Q RT SuperMix (Vazyme, China) to synthesize cDNA. The resultant cDNA served as a template for qPCR analysis on a StepOnePlus real-time fluorescence quantitative PCR system using ChamQ Universal SYBR qPCR Master Mix (Vazyme, China). The PCR cycling protocol consisted of pre-denaturation at 95°C for 3 min, 40 cycles of denaturation at 95°C for 10 s, annealing at 60°C for 15 s, extension at 60°C for 15 s, and a final extension at 60°C for 2 min. The expression levels of target mRNAs were calculated using the 2^-ΔΔCt^ method by normalizing to glyceraldehyde-3-phosphate dehydrogenase (GAPDH), and the corresponding primers are listed in Table 1.

**Table 1:** RT-qPCR primer.

Gene name	Sequences (5′ − 3′)	Accession number
GAPDH	F: TCTGACAGTCCGTCTTGAGAAA	NM_001115114.1
	R: ACAAAGTGATCGTTGAGAGAA	
PPAR-α	F: CGTCGTCAGGTGTTTACGGT	NM_001102567
	R: AGGCACTTCTGGAATCGACA	
Fasn	F: ATCTGTTCCTGTTCGATGGC	XM_005169478
	R: AGCATATCTCGGCTGACGTT	
SREBP-1	F: CATCCACATGGCTCTGAGTG	NM_001105129
	R: CTCATCCACAAAGAAGCGGT	
PPAR-γ	F: CTGCCGCATACACAAGAAGA	NM_131467
	R: TCACGTCACTGGAGAACTCG	
GPR43	F: CGTCGCATTTCCAATCCGAT	NM_001082895.1
	R: TCACATGGGGATTGAGCTGT	
Leptin A	F: CATCATCGTCAGAATCAGGG	NM_001128576
	R: ATCTCGGCGTATCTGGTCAA	

### 2.12 Detection of SCFAs in the culture supernatant of *Lactobacillus fermentum* E15

*Lactobacillus fermentum* E15 was inoculated into MRS broth and cultured at 37°C for 24 h, followed by centrifugation (10,000 rpm, 5 min) to collect the supernatant. Afterwards, 10 μL of internal standard (L-2-chlorophenylalanine, 0.3 mg/mL, prepared in methanol) was added into 100 μL of fermentation supernatant and vortexed for 10 s. 300 μL of methanol-acetonitrile (2:1, v/v) was added and vortexed for 1 min. Ultrasonic extraction was performed in ice water bath for 10 min and maintained at −20°C for 30 min. The supernatant was then collected by centrifugation at 13000 rpm, 4°C for 15 min. Next, 200 μL of the supernatant was transferred to an LC–MS/MS injection vial. The concentrations of SCFAs including acetic acid, propionic acid, isobutyric acid, butyric acid, 2-methylbutyric acid, isovaleric acid, valeric acid, 2,2-dimethylbutyric acid, 2-ethylbutyric acid, 3,3-dimethylbutyric acid, 2-methylhexanoic acid, 3-methylhexanoic acid, 4-methylhexanoic acid in the culture supernatant were analyzed using LC - MS/MS.

### 2.13 Detection of SCFAs of *Lactobacillus fermentum* E15 in zebrafish

A total of 400 wild-type AB strain zebrafish larvae at 5 dpf were randomly divided into four groups (100 larvae in each group) and incubated in 1 L tanks. There are four groups in this experiment: the normal diet group, the normal diet+ *L. fermentum* E15 group, the HCD group, the HCD + *L. fermentum* E15 group. Starting from 5 dpf, the normal diet group was fed a normal diet. The normal diet+ *L. fermentum* E15 group was fed a normal diet together with 1 × 10^6^ CFU/mL *L. fermentum* E15. The HCD group was fed a HCD. The HCD + *L. fermentum* E15 group was fed a HCD together with 1 × 10^6^ CFU/mL *L. fermentum* E15. Each group was fed an equal amount of feed, 30 mg/day (twice daily), and residual food was removed one hour after feeding. After 10 days, 20 zebrafish larvae in each group were randomly selected as one sample, and 3 samples were prepared for assessing SCFAs. Thirty zebrafish were put into a 1.5 mL EP tube with 400 μL of extract solution (methanol-acetonitrile-water = 2:2:1, v/v/v) and forty small steel balls. The EP tube was placed in the refrigerator at −80°C for 5 min, and then the contents of the EP tube were ground in the grinder (60 Hz, 2 min). The sample was centrifuged at low temperature for 15 min (13,000 rpm, 4°C) to collect the supernatant. The acetic acid, propionic acid, butyric acid, and isovaleric acid in the supernatant were detected using LC–MS/MS.

### 2.14 Effects of isovaleric acid on lipid accumulation and expression of GPR43 and leptin a mRNA in zebrafish

A total of 420 wild-type AB strain zebrafish larvae at 5 dpf were randomly divided into six groups (70 zebrafish larvae in each group) then incubated in 1 L water tanks. These six groups comprised a series of treatments, including the control group (fed a normal diet), the HCD group (fed a HCD), the *L. fermentum* E15 group (fed a HCD and 1 × 10^6^ CFU/mL *L. fermentum* E15), the *L. fermentum* E15+ pertussis toxin (PTX) group (fed a HCD, 1 × 10^6^ CFU/mL *L. fermentum* E15 and 50 ng/mL PTX), the isovaleric acid group (fed a HCD and 100 μM isovaleric acid), and the isovaleric acid + PTX group (fed a HCD, 100 μM isovaleric acid and 50 ng/mL PTX). After 5 dpf, the control group was fed a normal diet (30 mg/d, twice a day), while the other groups were fed a HCD (30 mg/d, twice a day) for 7 days (5–12 dpf). In the following 14-day period (12–26 dpf), the control group and HCD group continued to be fed as described above. Meanwhile, the *L. fermentum* E15 group was fed a HCD with the addition of *L. fermentum* E15 (30 mg/d, twice a day). The *L. fermentum* E15 + PTX group was fed a HCD, 1 × 10^6^ CFU/mL of *L. fermentum* E15, and 50 ng/mL of PTX. The isovaleric acid group was fed a HCD and 100 μM of isovaleric acid. The isovaleric acid+PTX group was fed a HCD, 100 μM of isovaleric acid, and 50 ng/mL PTX. All the groups had residual food removed after 1 h of feeding. After the intervention, the lipid accumulation in the zebrafish body was evaluated using Oil Red O staining. Meanwhile, the RT-qPCR was employed to analyze the expression of GPR43 and leptin A mRNA.

### 2.15 Statistical analysis

All the data were expressed as mean values ± standard deviation (SD), and the statistical analyses were performed using SPSS (version 20.0). One-way ANOVA followed by the Tukey multiple comparisons test was used for comparison between multiple groups. A *p* value<0.05 was considered as statistically significant. The graphs were obtained using GraphPad Prism 5 software.

## 3 Results

### 3.1 Morphological characteristics of *Lactobacillus fermentum* E15

*Lactobacillus fermentum* E15 grew well on MRS medium. After 48 h of incubation, the bacterial colonies appeared circular, with a milky, waxy, and white surface, with entire margin (Figure 1B). Gram staining confirmed that the strain is Gram-positive (Figure 1C).

### 3.2 *Lactobacillus fermentum* E15 can reduce lipid accumulation in the HCD-fed zebrafish larvae

**Figure 2:**
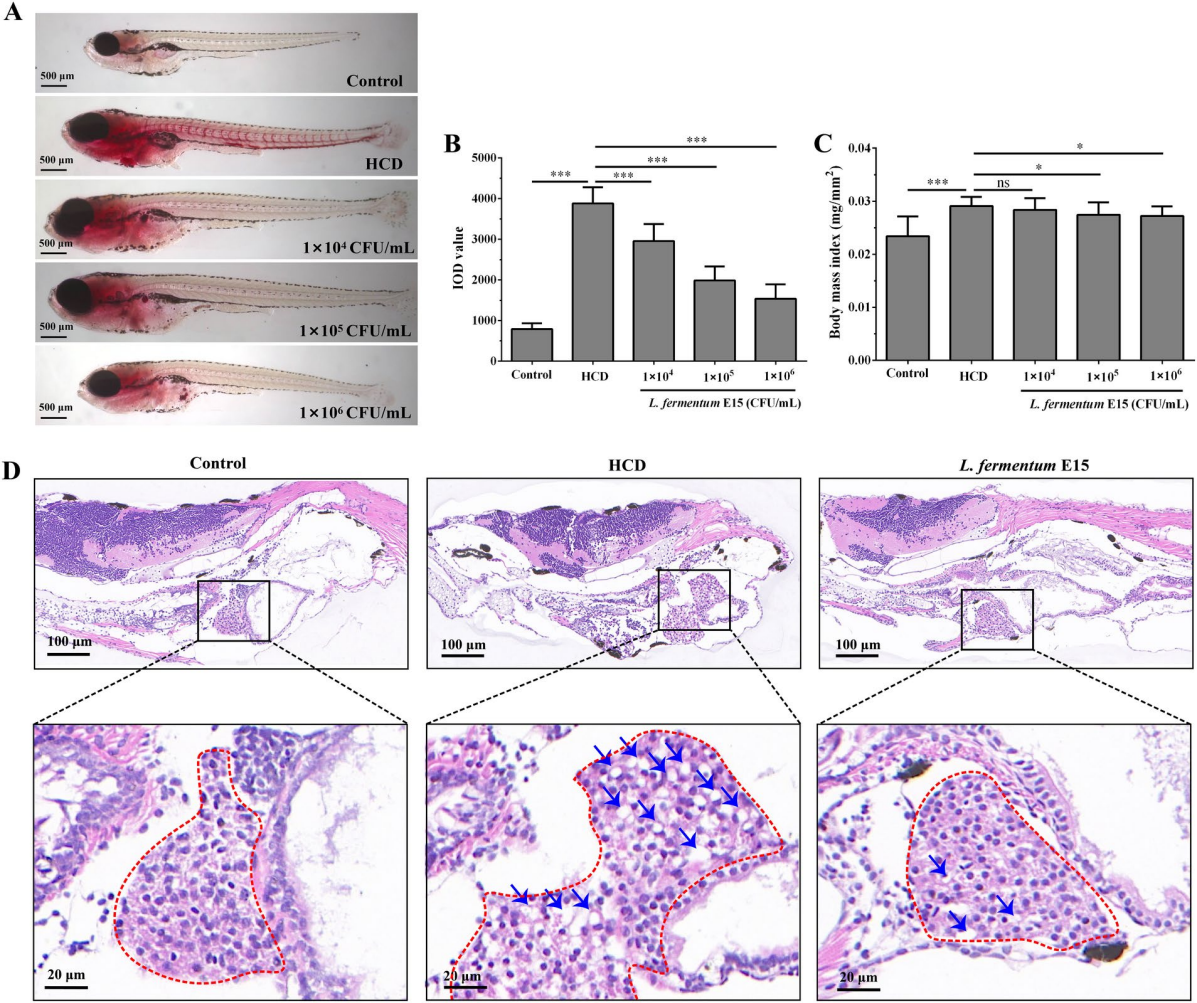
*Lactobacillus fermentum* E15 can improve lipid accumulation induced by a HCD in the liver and blood vessels of zebrafish. **(A)** Representative images of lipid accumulation in zebrafish using Oil Red O staining. **(B)** Zebrafish IOD values. The data are presented as mean values ± SD (*n* = 20). **(C)** Zebrafish BMI index. The data are presented as mean values ± SD (*n* = 20). **(D)** H&E staining of zebrafish liver paraffin sections (lipid droplets indicated by blue arrows). *p < 0.05, ***p < 0.001, and ns indicates that it is not statistically significant. *p* < 0.05 was considered as statistically significant calculated by One-way ANOVA followed by Tukey’s test.

No obvious lipid accumulation can be observed in the liver and blood vessels of the control group, whereas a substantial accumulation of lipid droplets can be detected in those of the HCD group (Figure 2A). Notably, the accumulation of red lipid droplets in the liver and blood vessels of zebrafish larvae treated with *L. fermentum* E15 was significantly reduced compared with the HCD group. Meanwhile, the DOI values in the *L. fermentum* E15 group (1 × 10^4^ CFU/mL group: 2955.18 ± 418.74; 1 × 10^5^ CFU/mL group: 1984.73 ± 344.58; 1 × 10^6^ CFU/mL group: 1536.17 ± 354.87) were significantly lower than those in the HCD group (3881.41 ± 397.32) (*p* < 0.001; Figure 2B). Additionally, the BMI was increased by HCD diet (0.029 ± 0.002 mg/mm^2^) as compared with the control group (0.023 ± 0.004 mg/mm^2^) (*p* < 0.001), suggesting that obesity was induced (Figure 2C, Table S1). The *L. fermentum* E15 treatment (1 × 10^5^ CFU/mL group: 0.027 ± 0.002 mg/mm^2^; 1 × 10^6^ CFU/mL group: 0.027 ± 0.001 mg/mm^2^) significantly inhibited the increase of BMI as compared to the HCD group (*p* < 0.05). The liver histopathological observations showed that the liver tissue of the control group was intact with dense arrangement of liver cells (Figure 2D). Meanwhile, many lipid vacuoles, deformed hepatocytes, and irregular arrangements in the liver tissue were observed in the HCD group, and the liver cells lost normal features with irregular arrangement. In contrast, there was a significant amelioration in the *L. fermentum* E15 (1 × 10^6^ CFU/mL) group, which showed a significant reduction of lipid vacuoles in the liver issue with normal morphology and arrangement of liver cells. These results suggested that *L. fermentum* E15 can remarkably improve the HCD diet-induced lipid accumulation in the liver and blood vessels.

### 3.3 *Lactobacillus fermentum* E15 can improve lipid metabolic disorder and liver function abnormalities in the HCD-fed zebrafish larvae

**Figure 3:**
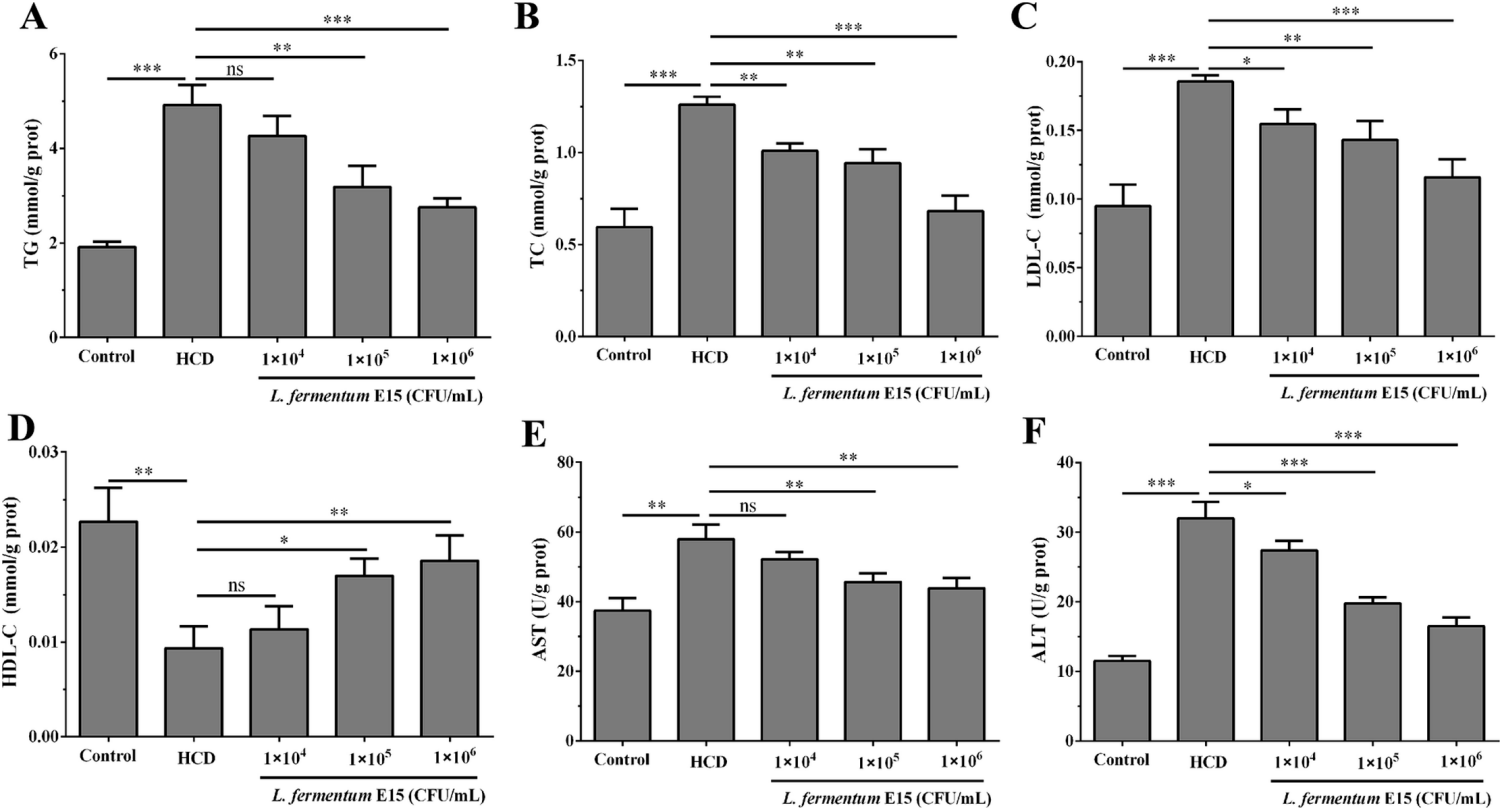
*Lactobacillus fermentum* E15 can improve lipid metabolic disorder and liver function abnormalities. The levels of **(A)** TC, **(B)** TG, **(C)** LDL-C, and **(D)** HDL-C in zebrafish larvae. Activities of **(E)** ALT and **(F)** AST in zebrafish larvae. The data are presented as mean values ± SD of three samples (*n* = 10 zebrfish for each sample). *p < 0.05, **p < 0.01, ***p < 0.001 and ns indicates that it is not statistically significant. *p* < 0.05 was considered as statistically significant calculated by One-way ANOVA followed by Tukey’s test.

The lipid-lowering effect of *L. fermentum* E15 was further evaluated by assessing the levels of TG, TC, LDL-C and HDL-C. As compared with the control group (TG: 1.910 ± 0.113 mmol/g prot; TC: 0.594 ± 0.100 mmol/g prot; LDL-C: 0.095 ± 0.016 mmol/g prot), higher levels of TG (4.916 ± 0.427 mmol/g prot), TC (1.260 ± 0.042 mmol/g prot) and LDL-C (0.186 ± 0.005 mmol/g prot) were detected in the HCD group (*p* < 0.001), while the level of HDL-C (0.009 ± 0.002 mmol/g prot) was significantly lower than in the control group (0.023 ± 0.004 mmol/g prot) (*p* < 0.01) (Figures 3A–D). After the intervention of *L. fermentum* E15, compared with the HCD group, the levels of TG (1 × 10^5^ CFU/mL group: 3.182 ± 0.446 mmol/g prot; 1 × 10^6^ CFU/mL group: 2.753 ± 0.186 mmol/g prot), TC (1 × 10^4^ CFU/mL group: 1.008 ± 0.041 mmol/g prot; 1 × 10^5^ CFU/mL group: 0.941 ± 0.076 mmol/g prot; 1 × 10^6^ CFU/mL group: 0.682 ± 0.083 mmol/g prot), and LDL-C (1 × 10^4^ CFU/mL group: 0.155 ± 0.011 mmol/g prot; 1 × 10^5^ CFU/mL group: 0.143 ± 0.014 mmol/g prot; 1 × 10^6^ CFU/mL group: 0.116 ± 0.013 mmol/g prot) were significantly reduced (*p* < 0.05), and the level of HDL-C (1 × 10^5^ CFU/mL group: 0.017 ± 0.002 mmol/g prot; 1 × 10^6^ CFU/mL group: 0.019 ± 0.003 mmol/g prot) was increased significantly (*p* < 0.05). Meanwhile, the biochemical indicators ALT and AST were also assessed to reflect liver function. The activity of ALT (31.972 ± 2.379 U/g prot) and AST (57.963 ± 4.214 U/g prot) in zebrafish larvae was significantly increased by feeding HCD as compared to the control group (ALT: 11.522 ± 0.737 U/g prot; AST: 37.432 ± 3.618 U/g prot) (*p* < 0.05). As compared to the HCD group, the ALT (1 × 10^4^ CFU/mL group: 27.405 ± 1.340 U/g prot; 1 × 10^5^ CFU/mL group: 19.764 ± 0.889 U/g prot; 1 × 10^6^ CFU/mL group: 16.508 ± 1.289 U/g prot) and AST (1 × 10^5^ CFU/mL group: 45.659 ± 2.560 U/g prot; 1 × 10^6^ CFU/mL group: 43.881 ± 2.971 U/g prot) activities of zebrafish larvae in *L. fermentum* E15 group were significantly decreased (*p* < 0.05), suggesting that *L. fermentum* E15 can ameliorate lipid metabolic disorder and liver function abnormalities (Figures 3E,F).

### 3.4 Effects of *Lactobacillus fermentum* E15 on genes related to lipid metabolism

**Figure 4:**
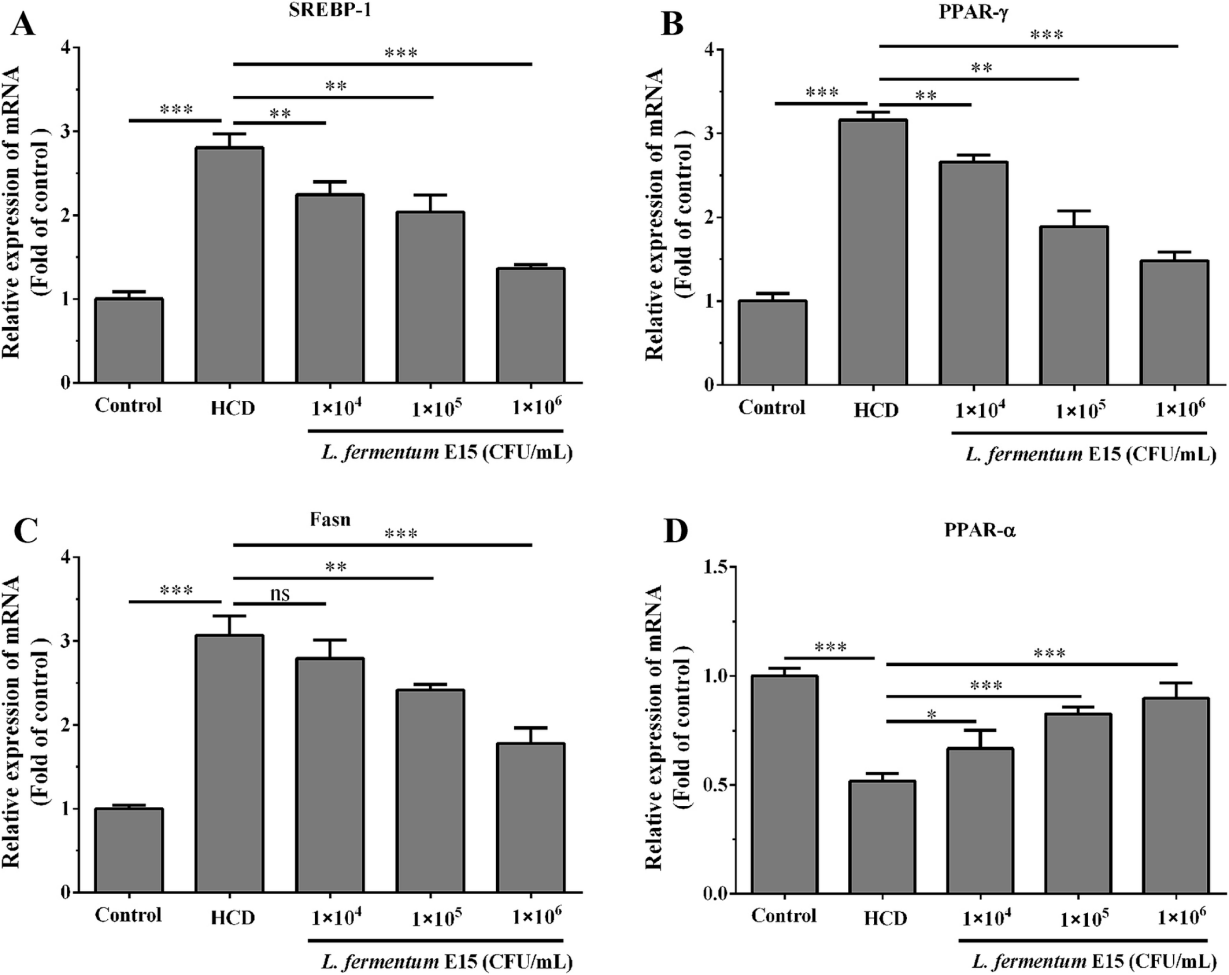
Expression levels of mRNA associated with lipid metabolism in zebrafish larvae regulated by *L. fermentum* E15. **(A)** mRNA expression level of SREBP-1. **(B)** mRNA expression level of PPAR-*γ*. **(C)** mRNA expression level of Fasn. **(D)** mRNA expression level of PPAR-*α*. The data are presented as mean values ± SD of three samples (*n* = 20 zebrfish for each sample). *p < 0.05, **p < 0.01, ***p < 0.001 and ns indicates that it is not statistically significant. *p* < 0.05 was considered as statistically significant calculated by One-way ANOVA followed by Tukey’s test.

To determine the association between the alterations in the aforementioned biochemical parameters and the alterations in gene expression, the mRNA expression levels of SREBP-1, PPAR-*γ*, Fasn, and PPAR-*α* lipid metabolism-related molecules were detected. The RT-qPCR analysis showed that compared with the control group, mRNA expression levels of SREBP-1 (2.81 fold), PPAR-γ (3.16 fold) and Fasn (3.07 fold) in HCD-induced zebrafish larvae were significantly increased (*p* < 0.001) (Figures 4A–C), while those of PPAR-*α* (0.52 fold) were significantly decreased (*p* < 0.001) (Figure 4D). In addition, the zebrafish in 1 × 10^4^ CFU/mL *L. fermentum* E15 group (SREBP-1: 2.24 fold; PPAR-γ: 2.66 fold), 1 × 10^5^ CFU/mL *L. fermentum* E15 group (SREBP-1: 2.04 fold; PPAR-γ: 1.89 fold), and 1 × 10^6^ CFU/mL *L. fermentum* E15 group (SREBP-1: 1.36 fold; PPAR-γ: 1.48 fold) showed a significant reduction in mRNA expression levels of SREBP-1 and PPAR-γ as compared to the HCD group (*p* < 0.01; Figures 4A,B). The mRNA expression levels of Fasn showed a significant reduction in the *L. fermentum* E15 group (1 × 10^5^ CFU/mL group: 2.41 fold; 1 × 10^6^ CFU/mL group: 1.78 fold) compared with the HCD group (*p* < 0.01) (Figure 4C). Moreover, the *L. fermentum* E15 groups had a significantly higher mRNA expression level of PPAR-α (1 × 10^4^ CFU/mL group: 0.67 fold; 1 × 10^5^ CFU/mL group: 0.83 fold; 1 × 10^6^ CFU/mL group: 0.90 fold) compared with the HCD group (*p* < 0.05; Figure 4D). These results showed that *L. fermentum* E15 probably alleviated HCD-induced hyperlipidemia by regulating lipid metabolism disorder.

### 3.5 *Lactobacillus fermentum* E15 can reduce oxidative damage in the HCD-fed zebrafish larvae

**Figure 5:**
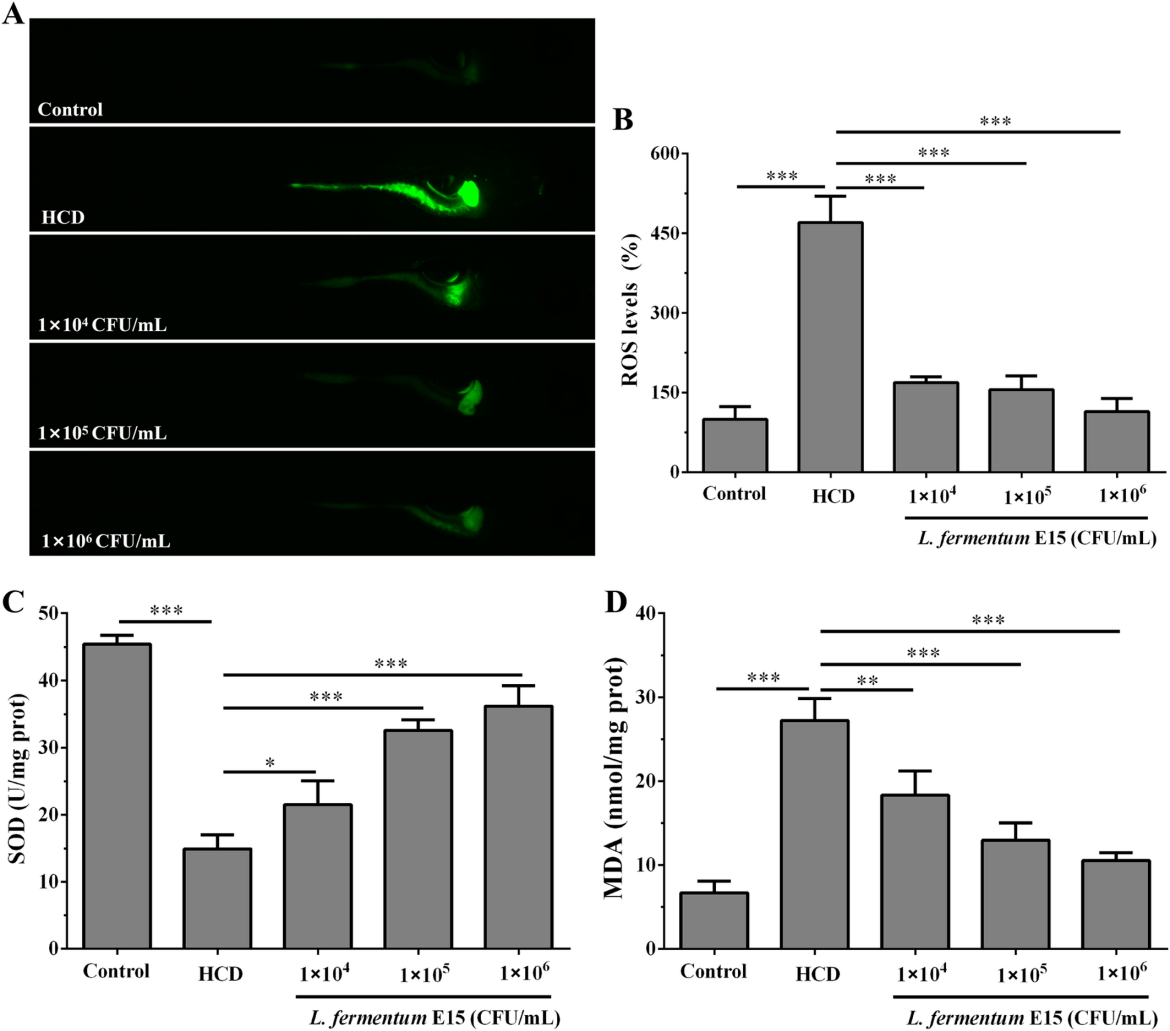
Effect of *L. fermentum* E15 on metabolic oxidative stress in HCD-fed zebrafish larvae. **(A)** Detection of ROS production in zebrafish larvae using DCFH-DA staining. **(B)** Quantification of ROS levels. The data are presented as mean values ± SD (*n* = 10). **(C)** SOD activity in zebrafish larvae. The data are presented as mean values ± SD of three samples (*n* = 10 zebrfish for each sample). **(D)** MDA levels in zebrafish larvae. The data are presented as mean values ± SD of three samples (*n* = 10 zebrfish for each sample). *p < 0.05, **p < 0.01, ***p < 0.001. *p* < 0.05 was considered as statistically significant calculated by One-way ANOVA followed by Tukey’s test.

High-fat dietary intake is highly associated with oxidative stress, including increased ROS production and severe oxidative damage to liver tissue (26). The fluorescence intensity of DCFH-DA staining showed that the ROS levels in zebrafish larvae in the HCD group (470.30 ± 49.57%) were significantly increased as compared to the control group (100.00 ± 23.98%) (*p* < 0.001; Figures 5A,B). The abdominal and liver tissues of zebrafish larvae in the HCD group showed an high ROS level, which was possibly attributed to the lipid accumulation in these regions. However, *L. fermentum* E15 significantly reduced the ROS level (1 × 10^4^ CFU/mL group: 169.10 ± 10.66%; 1 × 10^5^ CFU/mL group: 155.66 ± 26.03%; 1 × 10^6^ CFU/mL group: 114.14 ± 24.78%) in the HCD-fed zebrafish larvae as compared to the HCD group (*p* < 0.001; Figures 5A,B). Previous study showed that SOD belongs to an essential antioxidant enzyme that plays a critical role in mitigating the negative effects of free radicals in the body. Conversely, MDA is a cytotoxic molecule that induces cross-linking and polymerization of macromolecules including proteins (24). The effects of *L. fermentum* E15 on SOD activity and MDA levels in the HCD-fed zebrafish larvae were further investigated. As compared to the control group (SOD: 45.42 ± 1.31 U/mg prot; MDA: 6.69 ± 1.38 nmol/mg prot), a dramatic reduction of SOD activity (14.89 ± 2.09 U/mg prot) was detected in the HCD group (*p* < 0.001), while the MDA level (27.21 ± 2.65 nmol/mg prot) was significantly higher (*p* < 0.001; Figures 5C,D). The *L. fermentum* E15 group showed a significantly higher SOD activity (1 × 10^4^ CFU/mL group: 21.48 ± 3.54 U/mg prot; 1 × 10^5^ CFU/mL group: 32.54 ± 1.63 U/mg prot; 1 × 10^6^ CFU/mL group: 36.17 ± 3.06 U/mg prot) and a lower MDA level (1 × 10^4^ CFU/mL group: 118.34 ± 2.86 nmol/mg prot; 1 × 10^5^ CFU/mL group: 12.98 ± 2.04 nmol/mg prot; 1 × 10^6^ CFU/mL group: 10.54 ± 0.93 nmol/mg prot) as compared to the HCD group (*p* < 0.05). These results suggested that *L. fermentum* E15 can mitigate the oxidative damage in the HCD-fed zebrafish larvae.

### 3.6 *Lactobacillus fermentum* E15 can produce SCFAs

**Figure 6:**
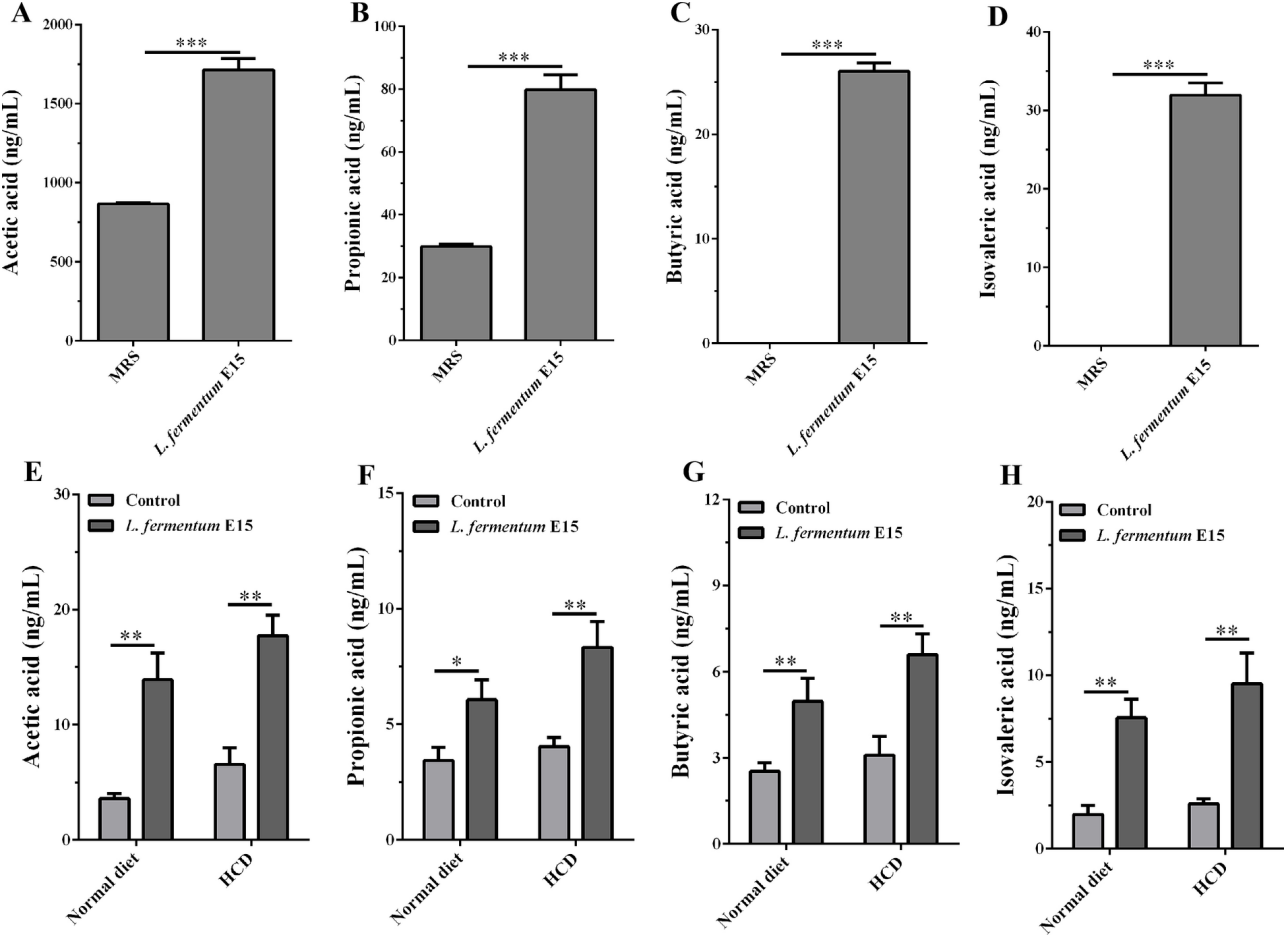
*Lactobacillus fermentum* E15 can produce SCFAs. The levels of **(A)** acetic acid, **(B)** propionic acid, **(C)** butyric acid, and **(D)** isovaleric acid in the culture supernatant of *L. fermentum* E15. The data are presented as mean values ± SD (*n* = 3). The levels of **(E)** acetic acid, **(F)** propionic acid, **(G)** butyric acid, and **(H)** isovaleric acid in zebrafish after supplementing with *L. fermentum* E15. The data are presented as mean values ± SD of three samples (*n* = 20 zebrfish for each sample). *p < 0.05, **p < 0.01, ***p < 0.001. *p* < 0.05 was considered as statistically significant calculated by One-way ANOVA followed by Tukey’s test.

SCFAs are the main metabolic products of gut microbiota that serve as an important energy source for colonic epithelial cells (27) and play a critical role in inhibiting the occurrence of obesity by regulating host energy metabolism and gut homeostasis (27, 28). Thus, the content of SCFAs in the culture supernatant of *L. fermentum* E15 was assessed using LC–MS/MS targeted metabolomics technology. The levels of acetic acid (1711.97 ± 72.02 ng/mL), propionic acid (79.68 ± 4.83 ng/mL), butyric acid (26.00 ± 0.80 ng/mL) and isovaleric acid (31.92 ± 1.57 ng/mL) in the culture supernatant of *L. fermentum* E15 were significantly increased as compared to the MRS medium group (acetic acid: 865.35 ± 7.09 ng/mL; propionic acid: 29.86 ± 0.83 ng/mL; butyric acid: 0 ng/mL; isovaleric acid: 0 ng/mL) (Figures 6A–D; *p* < 0.001). Additionally, we assessed the levels of acetic acid, propionic acid, butyric acid, and isovaleric acid in zebrafish larvae fed either a normal diet or a HCD. Our results showed that supplementation with *L. fermentum* E15 significantly increased the levels of acetic acid, propionic acid, butyric acid, and isovaleric acid both in zebrafish larvae fed either a normal diet or a HCD (Figures 6E–H) (*p* < 0.05).

### 3.7 *Lactobacillus fermentum* E15 can alleviate HCD-induced lipid accumulation via SCFAs-mediated activation of GPR43 receptor

**Figure 7:**
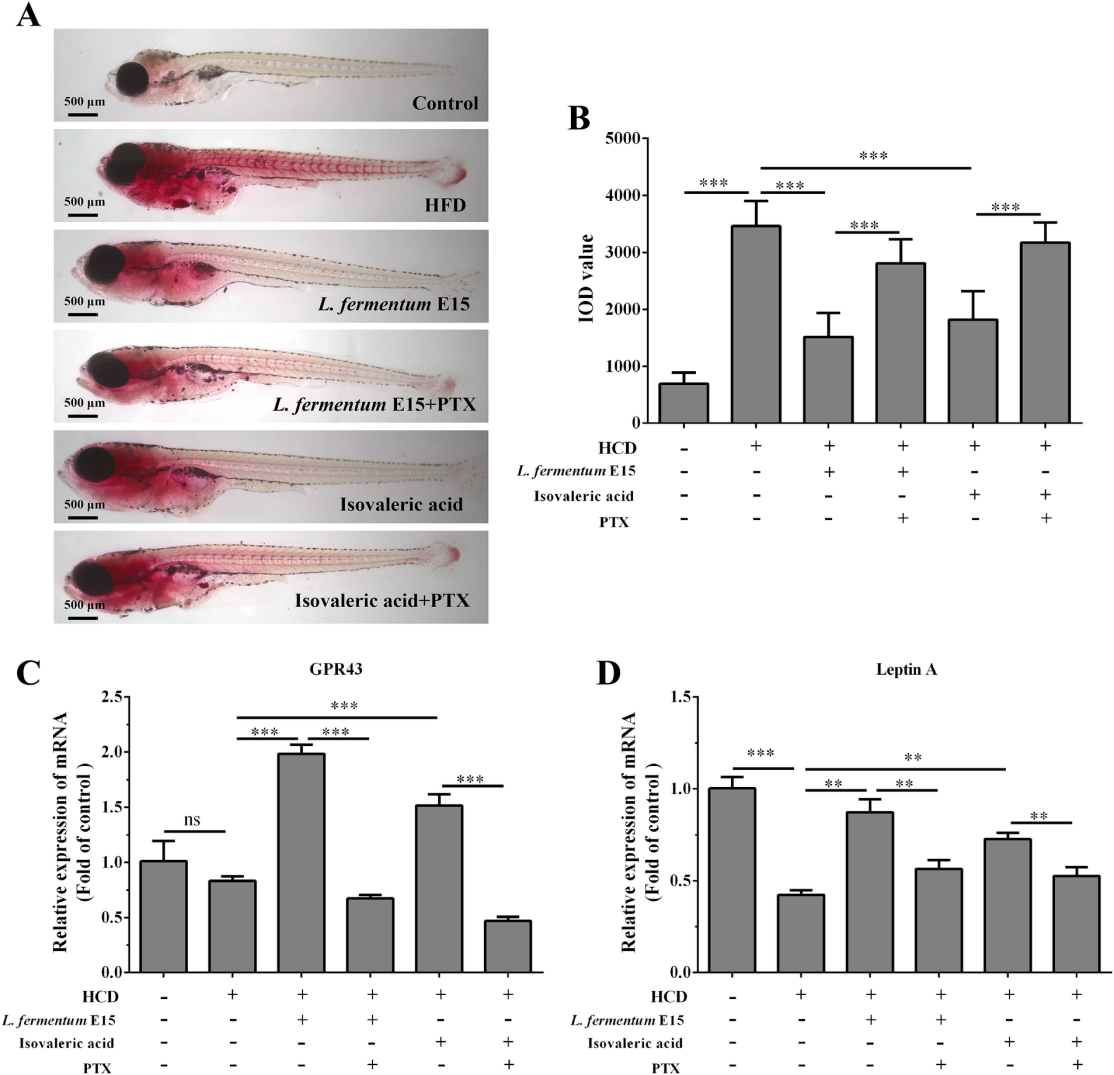
*Lactobacillus fermentum* E15 can alleviate HCD-induced lipid accumulation by activating the GPR43 receptor through the metabolism of SCFAs. **(A)** Representative images of lipid accumulation in zebrafish detected by Oil Red O staining. **(B)** IOD values in zebrafish. The data are presented as mean values ± SD (*n* = 20). The mRNA expression levels of GPR43 **(C)** and leptin A **(D)** detected by RT-qPCR. The data are presented as mean values ± SD of three samples (*n* = 20 zebrfish for each sample). *p < 0.05, **p < 0.01, ***p < 0.001 and ns indicates that it is not statistically significant. *p* < 0.05 was considered as statistically significant calculated by One-way ANOVA followed by Tukey’s test.

A recent study has reported that *Lactobacillus acidophilus* inhibits non-alcoholic fatty liver disease (NAFLD)-associated hepatocellular carcinoma through producing valeric acid (29). To shed light on the mechanisms by which *L. fermentum* E15 improves hyperlipidemia, the effects of isovaleric acid on lipid accumulation in HCD-fed zebrafish larvae were further investigated. The GPR43 receptor has been reported to be the primary receptor for SCFAs in intestine and liver (7, 29). To investigate whether the anti-hyperlipidemic effects of *L. fermentum* E15 are mediated by SCFAs, PTX was used to block the GPR43 receptor (29). A significantly lower accumulation of red lipid droplets was detected in the liver and blood vessels of zebrafish larvae from both the *L. fermentum* E15 group and the isovaleric acid group as compared to the HCD group (Figure 7A). In contrast, the effects of *L. fermentum* E15 and isovaleric acid on reducing lipid accumulation in HCD-fed zebrafish larvae were eliminated when the GPRs inhibitor (PTX) was used (Figures 7A,B). These findings suggested that isovaleric acid metabolized by *L. fermentum* E15 can reduce lipid accumulation in HCD-fed zebrafish larvae.

In order to investigate the mechanism behind the effects of *L. fermentum* E15 on HCD-induced hyperlipidemia in zebrafish larvae, the mRNA expression levels of GPR43 and leptin A were detected. Significantly increased mRNA expression levels of GPR43 and leptin A were observed in the *L. fermentum* E15 group (GPR43: 1.98 fold; leptin A: 0.87 fold) and isovaleric acid group (GPR43: 1.51 fold; leptin A: 0.73 fold) as compared to the HCD group (GPR43: 0.83 fold; leptin A: 0.42 fold) (*p* < 0.01; Figures 7C,D). In addition, the GPRs inhibitor PTX effectively inhibited the promoting effects of *L. fermentum* E15 and isovaleric acid on mRNA expression levels of GPR43 and leptin A (*p* < 0.01). These results suggested that *L. fermentum* E15 can alleviate HCD-induced lipid accumulation by activating the GPR43 receptor through the metabolism of SCFAs.

## 4 Discussion

Hyperlipidemia is mainly characterized by abnormal blood lipid metabolism, which can lead to various metabolism-related diseases such as atherosclerosis, diabetes and obesity (1, 2). Many studies have demonstrated a strong interaction between gut microbiota and hyperlipidemia along with its metabolic comorbidities (7, 9, 10). Nevertheless, the specific mechanism behind lipid-lowering effects of microorganisms remains unclear. In the present study, our objective was to investigate the potential hypolipidemic effect of *L. fermentum* E15 and the mechanisms underlying this effect. To achieve this, the anti-hyperlipidemic effects of *L. fermentum* E15 were assessed through Oil Red O staining, H&E staining, lipid concentrations (TG, TC, LDL-C, and HDL-C), and liver enzyme activities (AST and ALT). Additionally, the levels of SCFAs produced by *L. fermentum* E15 were measured using LC–MS/MS. To verify the hypothesis that the anti-hyperlipidemic effect of *L. fermentum* E15 occurs through SCFAs, we blocked GPR43 in zebrafish larvae using PTX to evaluate the alleviating effects of *L. fermentum* E15 and isovaleric acid on lipid metabolism disorders.

The main pathological change in hyperlipidemia is manifested as an abnormal serum lipid level. It should be noted that there is a significant positive correlation between the serum TC level and the occurrence of cardiovascular diseases (30). The increased TG, LDL-C, and reduced HDL-C are highly associated with metabolic diseases (31). Moreover, the liver is a key organ that regulates lipid metabolism including the metabolism of TG and TC (32, 33). In the case of hypercholesterolemia, excess lipids can accumulate in the form of lipid droplets in liver cells, leading to NAFLD and liver damage (34). Typically, ALT and AST are important indicators for evaluating liver damage (35). In the present study, it was found that *L. fermentum* E15 could effectively reduce lipid accumulation in blood vessels and liver of HCD-fed zebrafish larvae, decreasing BMI and hepatic lipid vacuoles (Figures 2A–D). Additionally, *L. fermentum* E15 treatment can prevent the HCD-induced abnormal levels of TC, TG, LDL-C and HDL-C (Figures 3A–D) and the increased activities of ALT and AST (Figures 3E,F). These results suggested that *L. fermentum* E15 can alleviate lipid accumulation in the liver and blood vessels of HCD-fed zebrafish larvae, and improve liver function. Mounting evidence indicates significant gender disparities in the incidence of hyperlipidemia, with women being more susceptible to dyslipidemia than men (36, 37). A previous study has suggested that 1-deoxynojirimycin exerts a remarkable female-preferential antihyperlipidemic effect through specifically enriching *Akkermansia* and *Clostridium* XIVa and elevating an active microbial metabolite in female mice (38). This finding suggests that gender disparities in regulating the gut microbiota could offer a novel strategy for developing next-generation antihyperlipidemic drugs (38). Therefore, further research is needed to explore gender differences in the hypolipidemic effect of *L. fermentum* E15 on adult zebrafish, which will provide an in-depth theoretical basis for therapeutic exploitation of *L. fermentum* E15 as a female-specific intervention against hyperlipidemia and provide a methodological reference for evaluating antihyperlipidemic drugs with gender differences. Previous study has confirmed that *Lactobacillus rhamnosus* GG can reduce hepatic lipid accumulation and suppress the progression of diet-induced liver steatosis (39). Besides, *Lactobacillus fermentum* HNU312 can ameliorate lipid accumulation in liver and adipose tissue of HCD-fed mice, thereby decreasing body weight and BMI. Moreover, *Lactobacillus fermentum* HNU312 can significantly reduce the levels of TG, TC, and LDL-C (40). These findings are consistent with our current results. However, further work is required to understand the action pathways and mechanisms of *Lactobacillus fermentum* HNU312.

PPAR-*γ* and SREBP-1 are transcription factors that promote lipid synthesis and storage in adipose tissue by positively regulating the expression of key lipogenic genes, such as Fasn (41, 42). In this work, HCD feeding caused hyperlipidemia, resulting in increased gene expression of SREBP-1, PPAR-γ and Fasn (Figures 4A–C). The reduced gene expressions of SREBP-1 and PPAR-γ might decrease lipid accumulation in the liver of hyperlipidemic zebrafish treated with *L. fermentum* E15. Previous studies have suggested that PPAR-*α* plays a role in fatty acid oxidation (35, 43). Therefore, a decrease in the expression of PPAR-α leads to dyslipidemia and lipid accumulation in the liver. The gene expression of PPAR-α was increased by *L. fermentum* E15 (Figure 4D), indicating that the fatty acid oxidation was enhanced. These results suggested that *L. fermentum* E15 might alleviate HCD-induced hyperlipidemia by regulating lipid metabolic disorders. Additionally, probiotics have the potential to exert persistent benefits after the cessation of treatment (44). Therefore, further study is needed to investigate the sustained anti-hyperlipidemia effects of *L. fermentum* E15 following treatment cessation.

Oxidative stress plays a critical role in the development of NAFLD (26, 45). HCD-induced cellular ROS overload can impair the antioxidant system of the liver, resulting to hepatocyte damage (46, 47). This imbalance in oxidative status can trigger lipid peroxidation, resulting in the production of MDA and excessive ROS, which further promotes the progression of hepatic steatosis (23). In this study, HCD feeding of zebrafish larvae resulted in the persistently high level of ROS, particularly observed around the abdomen and liver (Figure 5A), which correlated with lipid accumulation. It is widely believed that the accumulation of lipid droplets in the abdomen and liver is strongly associated with the increased production of ROS, which potentially leads to lipid peroxidation of liver tissue. The reduced SOD activity can disrupt cellular redox homeostasis to produce oxidative stress and damage. In the present case, *L. fermentum* E15 reduced the levels of ROS and MDA in HCD-fed zebrafish larvae, while enhancing SOD activity (Figures 5A–D). These results demonstrated that the antioxidant effect of *L. fermentum* E15 is favorable for protecting HCD-fed zebrafish larvae from the detrimental effects of NAFLD. Additionally, *Chaetoceros globosum* CGMCC 6882 decreased both H₂O₂-induced oxidative stress in HepG2 cells and lipopolysaccharide (LPS)-induced oxidative stress in RAW 264.7 cells (48, 49). Therefore, further research is needed to explore the inhibitory effect of *L. fermentum* E15 on LPS- or H_2_O_2_-induced oxidative stress.

One of the primary mechanisms by which the gut microbiota can influence host physiological functions is related to its metabolic activity (50, 51). Numerous studies have demonstrated that SCFAs produced by the gut microbiota act as an energy source for epithelial cells, signaling molecules and gene expression regulators, exerting significant impacts on host physiology (50–53). Gut microbiota has been reported to influence energy supply, blood glucose and lipid homeostasis through SCFAs like butyrate and propionate, thereby regulating the obesity-related physiological and pathological processes (54). A recent study has reported that *Lactiplantibacillus plantarum* A5 alleviates HCD-induced hyperlipidemia in hamsters by modulating the gut microbiota to enhance the production of SCFAs (55). Additionally, the correlation analysis has indicated that *Lactiplantibacillus plantarum* Y44 complex fermented milk influences hepatic lipid metabolism in HCD-fed C57BL/6 mice by regulating the intestinal flora and promoting SCFA production, ultimately contributing to weight reduction (56). Furthermore, *Lactobacillus acidophilus* has been reported to inhibit NAFLD-associated hepatocellular carcinoma through the production of valeric acid (29). Taken together, these findings suggest that *Lactobacillus* spp. may play a role in mitigating HCD-induced hyperlipidemia by promoting the production of SCFAs. In this study, targeted metabolomics analysis was employed to identify the SCFAs metabolized by *L. fermentum* E15 *in vitro*, which were identified as acetic acid, propionic acid, butyric acid and isovaleric acid (Figures 6A–D). Furthermore, in the presence of *L. fermentum* E15, the levels of acetic acid, propionic acid, butyric acid, and isovaleric acid increased in both zebrafish larvae fed a normal diet and those fed an HCD (Figures 6E–H). SCFAs can induce the release of hormones by binding to GPRs, thereby increasing satiety and reducing the food intake (7). Additionally, the increased leptin level can modulate lipid metabolism through signaling regulation (57, 58). The combination of SCFAs, such as butyrate and propionate, with GPR43 suppresses fatty acid breakdown, promotes the secretion of the well-known adipokine leptin, increases energy expenditure and inhibits adipocyte synthesis, thereby reducing lipid metabolism in mice (59). Notably, these effects are absent in GPR43 knockout mice (59). To explore the mechanisms by which *L. fermentum* E15 influences HCD-induced hyperlipidemia in zebrafish larvae, GPRs antagonist PTX was used to block GPR43 to verify the anti-hyperlipidemic effects of *L. fermentum* E15 and isovaleric acid. These findings suggested that *L. fermentum* E15 and isovaleric acid can reduce the accumulation of red lipid droplets in the liver and blood vessels of HCD-fed zebrafish larvae (Figures 7A,B). However, PTX could eliminate the effects of *L. fermentum* E15 and isovaleric acid on reducing lipid accumulation in HCD-fed zebrafish larvae. Additionally, both *L. fermentum* E15 and isovaleric acid enhanced the mRNA expression levels of GPR43 and leptin A (Figures 7C,D). However, PTX significantly inhibited the ability of *L. fermentum* E15 and isovaleric acid to enhance mRNA expression of GPR43 and leptin A. SCFA acts on GPR43 to mediate leptin response to control the cholesterol levels (58). Leptin has been proposed as a signaling molecule that reduces hepatic lipogenesis and cholesterol synthesis by inhibiting the expression of SREBP-1 and cholesterol-related genes, thereby reducing cholesterol levels and alleviating hepatic steatosis (60, 61). These findings indicated that *L. fermentum* E15 alleviates HCD-induced hyperlipidemia in zebrafish larvae by activating GPR43 through SCFAs. However, *Lactobacillus rhamnosus* TR08 has been shown to improve the intestinal microbiota of mice to increase the production of SCFAs, and then improve dyslipidemia (62). *Lactiplantibacillus plantarum* A5 alleviated HCD-induced hyperlipidemia *via* regulating gut microbiota to promote SCFAs production (55). Therefore, we will explore the potential of *L. fermentum* E15 to mediate changes in microbiome composition to promote the production of SCFAs in future work. In addition, previous studies have demonstrated that *Lactobacillus plantarum* can induce changes in food components, and then the active components in foods are absorbed by the body and exert bioactivity (63–65). Therefore, we will explore the potential of *L. fermentum* E15 to mediate changes in food components and evaluate whether the resulting active components can exert biological activity in future work.

In summary, the present study reveals that *L. fermentum* E15 can activate the GPR43 receptors through the metabolism of SCFAs to regulate the gene expression of lipid metabolism-related factors. As a result, *L. fermentum* E15 ameliorates obesity in HCD-fed zebrafish larvae, reduces the lipid accumulation, and consequently decreases oxidative stress and liver damage.

## Data Availability

The original contributions presented in the study are included in the article/Supplementary material, further inquiries can be directed to the corresponding author/s.
