# Supplementary material for: Alleviating effect of Lactobacillus fermentum E15 on hyperlipidemia and hepatic lipid metabolism in zebrafish fed by a high-fat diet through the production of short-chain fatty acids
Source: Front Nutr. 2025 Mar 3;12:1522982. doi: 10.3389/fnut.2025.1522982 (PMC11911183; doi:10.3389/fnut.2025.1522982)
Supplement: Supplementary file 1 [file Table_1.docx]

**Supporting information**

**Alleviating effect of *Lactobacillus fermentum* E15 on hyperlipidemia and hepatic lipid metabolism in zebrafish fed by a high-fat diet through the production of short-chain fatty acids**

Yishu Chen^1,#^, Kangdi Zheng^2,#^, Yang Leng^1,#^, Zhao Zhang^1,2^, Xiaoling Li^1^, Xiaoyan Li^1^, Huajun Ou^1^, Muhao Wen^3^, Feng Qiu^3,*^, Huajun Yu^1,*^

^1^Laboratory Animal Center, Guangdong Medical University, Zhanjiang, 524023, China

^2^Guangdong Longseek Testing Co., Ltd., Guangzhou, Guangdong, 510700, China

^3^Department of Laboratory Medicine, the Seventh Affiliated Hospital of Southern Medical University, Foshan, Guangdong 528244, China

^#^These authors contributed equally.

*To whom correspondence should be addressed: Prof. Feng Qiu, E-mail: [qiufeng3079@smu.edu.cn;](mailto:qiufeng3079@smu.edu.cn;) Hua-Jun Yu, E-mail: [hjyu@gdmu.edu.cn](mailto:hjyu@gdmu.edu.cn)

**Table S1** Body length and weight of zebrafish (mean±SD, n=20)

| Groups | Body length (mm) | Weight (mg) | BMI (mg/mm^2^) |
| --- | --- | --- | --- |
| Control | 4.89 | 0.64 | 0.0268 |
|  | 5.17 | 0.69 | 0.0258 |
|  | 5.81 | 0.74 | 0.0219 |
|  | 5.11 | 0.6 | 0.0230 |
|  | 5.05 | 0.55 | 0.0216 |
|  | 5.06 | 0.54 | 0.0211 |
|  | 5.23 | 0.66 | 0.0241 |
|  | 5.26 | 0.59 | 0.0213 |
|  | 5.17 | 0.62 | 0.0232 |
|  | 5.21 | 0.56 | 0.0206 |
|  | 5.39 | 0.53 | 0.0182 |
|  | 5.55 | 0.54 | 0.0175 |
|  | 5.43 | 0.56 | 0.0190 |
|  | 5.32 | 0.51 | 0.0180 |
|  | 5.5 | 0.86 | 0.0284 |
|  | 5.7 | 0.89 | 0.0274 |
|  | 5.63 | 0.8 | 0.0252 |
|  | 5.82 | 0.94 | 0.0278 |
|  | 6.18 | 1.11 | 0.0291 |
|  | 6.17 | 1.07 | 0.0281 |
| HCD | 6.1 | 1.01 | 0.0271 |
|  | 5.41 | 0.9 | 0.0308 |
|  | 5.46 | 0.95 | 0.0319 |
|  | 5.64 | 0.93 | 0.0292 |
|  | 6.13 | 1.14 | 0.0303 |
|  | 5.44 | 0.84 | 0.0284 |
|  | 5.62 | 0.87 | 0.0275 |
|  | 6.00 | 0.99 | 0.0275 |
|  | 5.78 | 0.92 | 0.0275 |
|  | 5.34 | 0.90 | 0.0316 |
|  | 5.58 | 0.86 | 0.0276 |
|  | 5.43 | 0.89 | 0.0302 |
|  | 6.37 | 1.12 | 0.0276 |
|  | 5.46 | 0.92 | 0.0309 |
|  | 6.09 | 1 | 0.0270 |
|  | 6.1 | 1.07 | 0.0288 |
|  | 6.12 | 1.09 | 0.0291 |
|  | 6.25 | 1.18 | 0.0302 |
|  | 6.3 | 1.27 | 0.0320 |
|  | 5.84 | 0.92 | 0.0270 |
| 1×10^4^ CFU/mL | 6.04 | 1.05 | 0.0288 |
|  | 5.5 | 0.85 | 0.0281 |
|  | 6.15 | 1.27 | 0.0336 |
|  | 5.55 | 0.83 | 0.0269 |
|  | 6.21 | 1.26 | 0.0327 |
|  | 6.09 | 1.03 | 0.0278 |
|  | 5.99 | 1.00 | 0.0279 |
|  | 5.58 | 0.81 | 0.0260 |
|  | 5.48 | 0.84 | 0.0280 |
|  | 5.92 | 0.97 | 0.0277 |
|  | 5.79 | 0.94 | 0.0280 |
|  | 5.41 | 0.81 | 0.0277 |
|  | 5.77 | 0.96 | 0.0288 |
|  | 6.11 | 1.10 | 0.0295 |
|  | 5.97 | 0.98 | 0.0275 |
|  | 5.45 | 0.79 | 0.0266 |
|  | 5.44 | 0.73 | 0.0247 |
|  | 6.04 | 1.04 | 0.0285 |
|  | 5.89 | 0.93 | 0.0268 |
|  | 6.39 | 1.31 | 0.0321 |
| 1×10^5^ CFU/mL | 6.12 | 0.94 | 0.0251 |
|  | 5.42 | 0.76 | 0.0259 |
|  | 6.02 | 0.99 | 0.0273 |
|  | 6.16 | 1.19 | 0.0314 |
|  | 5.87 | 0.81 | 0.0235 |
|  | 5.63 | 0.93 | 0.0293 |
|  | 5.66 | 0.88 | 0.0275 |
|  | 6.44 | 1.21 | 0.0292 |
|  | 5.60 | 0.76 | 0.0242 |
|  | 5.54 | 0.83 | 0.0270 |
|  | 5.49 | 0.79 | 0.0262 |
|  | 6.01 | 1.06 | 0.0293 |
|  | 5.84 | 0.89 | 0.0261 |
|  | 6.14 | 1.07 | 0.0284 |
|  | 5.79 | 0.85 | 0.0254 |
|  | 6.06 | 1 | 0.0272 |
|  | 5.43 | 0.99 | 0.0336 |
|  | 5.49 | 0.81 | 0.0269 |
|  | 5.84 | 0.92 | 0.0270 |
|  | 5.77 | 0.95 | 0.0285 |
| 1×10^6^ CFU/mL | 6.38 | 1.14 | 0.0280 |
|  | 6.3 | 1.11 | 0.0280 |
|  | 6.2 | 1.16 | 0.0302 |
|  | 5.88 | 1.02 | 0.0295 |
|  | 6.03 | 1.04 | 0.0286 |
|  | 5.66 | 0.86 | 0.0268 |
|  | 5.54 | 0.80 | 0.0261 |
|  | 5.43 | 0.79 | 0.0268 |
|  | 5.80 | 0.76 | 0.0226 |
|  | 5.94 | 0.87 | 0.0247 |
|  | 6.06 | 0.97 | 0.0264 |
|  | 6.29 | 1.05 | 0.0265 |
|  | 6.07 | 1.02 | 0.0277 |
|  | 5.59 | 0.86 | 0.0275 |
|  | 5.47 | 0.80 | 0.0267 |
|  | 6.12 | 1.01 | 0.0270 |
|  | 5.68 | 0.99 | 0.0307 |
|  | 5.89 | 0.96 | 0.0277 |
|  | 5.94 | 0.91 | 0.0258 |
|  | 5.63 | 0.86 | 0.0271 |
